# Night shift work increases the risk of developing irritable bowel syndrome: a prospective cohort study in the UK Biobank

**DOI:** 10.3389/fpubh.2025.1651752

**Published:** 2025-10-16

**Authors:** Shiwei Lu, Laifu Li, Yan Zhuang, Fangchen Ye, Xinping Zhang, Jiamiao Chen, Zhuoya Sun, Fei Dai

**Affiliations:** Department of Gastroenterology, Second Affiliated Hospital of Xi'an Jiaotong University, Xi'an, China

**Keywords:** irritable bowel syndrome, night shift work, circadian rhythm, body mass index, cohort study, UK Biobank

## Abstract

**Background:**

Irritable bowel syndrome (IBS) is a functional gastrointestinal disorder associated with a substantial disease burden. Night shift work has become increasingly common and is related to various human diseases. This study investigates the relationship between night shift work and the risk of incident IBS.

**Methods:**

266,605 participants from the UK Biobank were included in our analysis. Data on shift work patterns, IBS incidence, and relevant covariates were obtained from the UK Biobank. Cox proportional hazard regression models were employed to assess the association between night shift work and IBS risk. Sensitivity analysis and subgroup analysis stratified by specific covariates were conducted to evaluate the robustness of the findings.

**Results:**

During a median follow-up of 9.03 years, 5,218 new incident IBS cases were identified. Compared to individuals who never/rarely engaged in night shift work, those who always worked the night shift were associated with an elevated risk of IBS across all models. Specifically, the hazard ratio and 95% confidence interval were 1.41 (1.23–1.60) for Model 1, 1.53 (1.35–1.76) for Model 2, and 1.36 (1.19–1.56) for the fully adjusted Model 3. These results remained consistent in the sensitivity analysis. Subgroup analysis revealed that the increased risk of IBS associated with always night shifts persisted across different genders, age groups, sleep durations, and mental health statuses. However, this association was only observed in individuals with a body mass index (BMI) ≥ 25 kg/m^2^.

**Conclusion:**

Individuals who always worked night shifts exhibited a higher risk of developing IBS compared to those who never/rarely engaged in night shift work.

## 1 Introduction

Irritable Bowel Syndrome (IBS) is a functional gastrointestinal disorder characterized by recurring abdominal pain connected to defecation or alterations in bowel habits. This condition typically manifests with irregular bowel habits such as constipation, diarrhea, or alternation of both and is along with abdominal bloating or distension. For clinical diagnosis, symptoms must appear at least 6 months before assessment and persist with active manifestations during the 3 months preceding diagnosis (1, 2). Epidemiological studies indicate that the global prevalence of irritable bowel syndrome exhibits significant geographical variations, ranging from 0.2 to 7.6% across different populations (3). This functional gastrointestinal disorder not only substantially impairs health-related quality of life (HRQoL) through chronic symptom burden but also reduces occupational productivity, ultimately imposing substantial disease burden on affected individuals through both direct healthcare utilization and indirect socioeconomic costs (4, 5). The pathophysiological mechanisms of IBS are complex and still under investigation. Possible mechanisms include visceral hypersensitivity (1), gut microbiota dysbiosis (6, 7), dysfunctional brain-gut axis (8), neurostructural modifications (9), low-grade inflammation (10), impaired intestinal barrier (11), gene (12), and neuroendocrine abnormalities (13).

Circadian rhythm, also known as the biological clock, refers to the periodic changes in physiology and behavior that occur in organisms due to the Earth’s rotation and the alternation of day and night (14). This complex phenomenon involves multiple mechanisms and exerts significant influences on various aspects such as metabolism, hormone secretion, and adaptive immunity within the organism (15–17). Most of the workers in Europe work between 08:00 and 19:00 from Monday to Friday, but an increasing number of workers do not follow these office working hours. Shift work is defined in Europe as working outside office working hours on different shift patterns, whereas night work comprises working at least 2 h between 22:00 and 05:00. The definitions of shift work vary a lot between surveys and countries (18). Some research indicates that shift work can increase the risk of developing metabolic syndrome (19), obesity (20), hypertension (21), impairing glucose tolerance (22), and cardiovascular diseases (23).

To date, several studies have examined the association between shift work and irritable bowel syndrome, however, these investigations are limited by small sample sizes and specific occupational cohorts (24, 25). There is a compelling need for a comprehensive, large-scale, population-based cohort study to elucidate the relationship between shift work and IBS. The objective of this study is to use the UK Biobank to construct a robust cohort model to assess the impact of shift work on the incidence of IBS.

## 2 Methods

### 2.1 Study design

UK Biobank is a large-scale database comprising over 500,000 participants recruited across England, Scotland, and Wales. Initiated between 2006 and 2010, the study conducted comprehensive baseline assessments through 22 dedicated research centers, systematically collecting multidimensional data, including demographic information, physiological measurements, lifestyle questionnaires, and biological samples. With rigorous ethical oversight and participant consent, this longitudinal initiative maintains ongoing follow-up through linkage to national health registries and periodic data updates (26).

**Figure 1:**
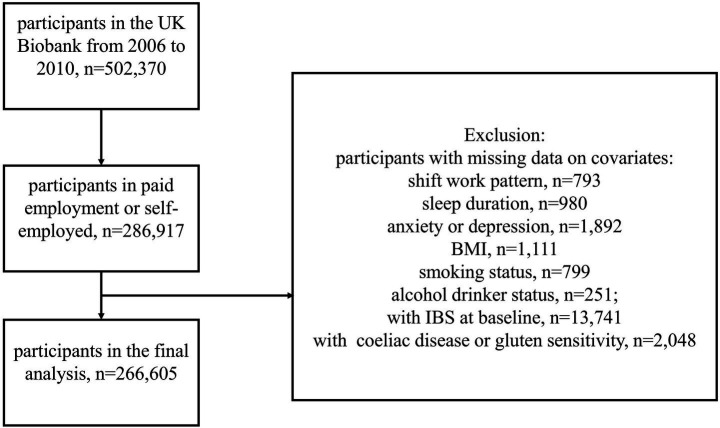
Flowchart of the study. BMI, Body mass index; IBS, Irritable bowel syndrome.

Our study primarily focused on participants who reported their current employment status as “In paid employment or self-employed” (Data-Field 6,142) in the UK Biobank. To mitigate reverse causality, control for confounding factors, and enhance statistical power, we excluded participants with a baseline diagnosis of IBS. Participants with common benign digestive system disorders demonstrating IBS-like symptoms were also excluded, including those with coeliac disease or gluten sensitivity (27, 28). Additionally, individuals with missing data on covariates (shift work pattern, sleep duration, anxiety, depression, body mass index, smoking status, and alcohol drinker status) were also excluded. After applying these exclusion criteria, a total of 266,605 participants met the requirements for inclusion in our analysis (Figure 1).

### 2.2 Shift work assessment

All participants underwent an occupational status assessment, during which they were asked, “Does your work involve shift work?” (Data-Field 826). Shift work is a work schedule that falls outside the normal daytime working hours of 9 a.m.–5 p.m. This may involve working afternoons, evenings, or nights or rotating through these kinds of shifts. If the answer was affirmative, they were further queried “Does your work involve night shifts?” (Data-Field 3,426). Night shifts are a work schedule that involves working through the normal sleeping hours, for instance, working through the hours from 12 a.m. to 6 a.m. Based on their responses to these two questions, participants were categorized into three groups: “Never/rarely night shifts “, “Sometimes/usually night shifts,” and “Always night shifts.” This classification allowed us to stratify the cohort according to the frequency and nature of their shift work exposure.

### 2.3 Outcome ascertainment

The diagnosis of IBS and the corresponding diagnosis dates were obtained from multiple sources, including self-reported medical conditions, primary care data, death register records, and hospital inpatient data. These data were linked to the UK National Health Services (NHS) register using the International Classification of Diseases, Version 10 (ICD-10) code K58 to diagnosis, which specifically pertains to irritable bowel syndrome.

This comprehensive approach ensured a robust and accurate identification of IBS cases within the study cohort. The date of death was obtained through linkage to national death registries (Data-Field 40,000). The end of follow-up was described as the date of the last personal contact with the UK Biobank (Data-Field 20,143). The study endpoint was defined as the occurrence of IBS, death, or the end of follow-up, whichever occurred first.

### 2.4 Covariates

In our study, we collected a range of sociodemographic characteristics, lifestyle habits, and health status as covariates. Sociodemographic characteristics include enrollment age (years), sex (male/female), ethnicity (white/non-white), and body mass index (BMI, kg/m^2^). Lifestyle habits include smoking status (never/previous/current smoker), alcohol drinker status (never/previous/current drinker), and sleep duration (average hours of sleep per day). Health status involves self-reported anxiety and depression (yes/no). These covariates were included in the analysis to adjust for potential confounders and to ensure a more accurate assessment of the relationship between night shift work and IBS. Data on these variables were obtained from self-reported questionnaires, primary care records, and linked health registries within the UK Biobank.

### 2.5 Statistical analysis

The baseline characteristics of the study participants were presented as mean ± standard deviation for continuous variables and number (percentage) for categorical variables. Cox proportional hazard regression models were employed to investigate the association between night shift work and the risk of IBS onset, with results expressed as hazard ratios (HRs) and 95% confidence intervals (CIs) (29). Night shift work patterns were categorized into three groups: “Never/rarely night shifts,” “Sometimes/usually night shifts,” and “Always night shifts.” The follow-up period referred to the time between recruitment and the study endpoint.

In the primary analysis, we explored the risk of developing IBS among participants who were “Sometimes/usually night shifts” or “Always night shifts” compared to those who were “Never/rarely night shifts,” using different research models. Model 1 examined the crude association between night shift work and IBS. Model 2 was adjusted for age and sex based on Model 1. Model 3 further used adjustments for sleep duration, anxiety, depression, BMI, smoking status, and alcohol drinker status based on Model 2. In all models, “Never/rarely night shifts” were set as the reference.

To investigate the heterogeneity in the impact of night shift work on the risk of developing IBS across different populations, thereby enabling early interventions for high-risk groups. We conducted a subgroup analysis by sex (male/female), age (<50 years old/≥50 years old), sleep duration (<7 h/≥7 h), BMI (<25 kg/m^2^/≥25 kg/m^2^), and anxiety or depression (yes/no). To mitigate the risk of multiple false positives, subgroup findings were considered exploratory and will require validation in future studies.

To enhance the robustness of our study findings, we conducted a sensitivity analysis by excluding IBS cases that occurred within 1 year after enrollment. This approach aimed to minimize the potential influence of reverse causality and ensure that the observed associations between night shift work and IBS were not driven by pre-existing or early-onset conditions that might have been undetected at baseline. Additionally, we further included residential address (urban/rural) and physical activity (low/moderate/high) as covariates to validate the robustness of the study findings.

All analyses were performed using R software, version 4.3.3. All *p* values were two-sided, and *p* < 0.05 was considered to indicate statistical significance.

## 3 Results

The baseline characteristics of the study participants are presented in Table 1. 266,605 individuals were included in the study. We found that night shift workers were more likely to be male, younger, non-white, have a higher index of multiple deprivation and BMI, be current smokers, and never consume alcohol.

**Table 1:** Baseline characteristics of the study participants in the UK Biobank.

Variables	Shift work patterns			*p*-value
	Never/rarely night shifts	Sometimes/usually night shifts	Always night shifts	
Number of participants	243,021	13,249	10,335	
Male, No, (%)	116,309 (47.9)	8,332 (62.9)	6,516 (63.0)	<0.001
Age, mean, (SD), years	52.92 (7.11)	51.20 (6.87)	51.32 (6.82)	<0.001
White, No, (%)	229,623 (94.5)	11,610 (87.6)	8,948 (86.6)	<0.001
Index of Multiple Deprivation, mean, (SD)	15.41 (13.37)	19.46 (15.94)	20.93 (16.58)	<0.001
Anxiety or depression, No, (%)	75,840 (31.2)	3,849 (29.1)	2,970 (28.7)	<0.001
BMI, mean, (SD), kg/m^2^	27.17 (4.66)	28.21 (4.87)	28.43 (4.90)	<0.001
Sleep duration, mean, (SD), hours	7.06 (0.95)	6.91 (1.07)	6.82 (1.19)	<0.001
Smoking status				<0.001
Never, No, (%)	140,411 (57.8)	6,983 (52.7)	5,470 (52.9)	
Previous, No, (%)	77,843 (32.0)	4,111 (31.0)	3,109 (30.1)	
Current, No, (%)	24,767 (10.2)	2,155 (16.3)	1,756 (17.0)	
Alcohol drinker status				<0.001
Never, No, (%)	7,676 (3.2)	610 (4.6)	629 (6.1)	
Previous, No, (%)	6,286 (2.6)	425 (3.2)	387 (3.7)	
Current, No, (%)	229,059 (94.3)	12,214 (92.2)	9,319 (90.2)	

BMI, Body mass index; SD, standard deviation.

Values are given as mean (standard deviation) for continuous variables and number (percentage) for categorical variables.

*p* for values of continuous variables were estimated by one-way ANOVA test.

*p* for values of categorical variables were estimated by chi-square test.

During a median follow-up period of 9.03 years, 5,218 new cases of IBS were recorded. The source of incident IBS cases is detailed in Supplementary Table 1. As presented in Table 2, among participants who never or rarely worked night shifts, there were 4,775 incident cases of IBS (4,775/243,021). In contrast, those who sometimes or usually worked night shifts reported 216 incident cases (216/13,249), while those who always worked night shifts had 227 incident cases (227/10,335). We found that in the unadjusted Model 1, participants who always worked night shifts had a higher risk of developing IBS compared to those who never/rarely worked night shifts, with an HR of 1.41 (1.23–1.60). This result remained robust in Model 2 and Model 3. In Model 2 (adjusted for age and sex), the HR was 1.53 (1.35–1.76), and in the fully adjusted Model 3, it was 1.36 (1.19–1.56). However, we observed that in all models, the risk of developing IBS among participants who sometimes/usually worked night shifts did not significantly change compared to those who never/rarely worked night shifts. The results were not statistically significant.

**Table 2:** Association between night shift work and irritable bowel syndrome.

Variables	Never/rarely night shifts	Sometimes/usually night shifts	*p*-value	Always night shifts	*p*-value
Case/all subjects	4,775/243,021	216/13,249		227/10,335	
Model 1	1.00 (reference)	0.98 (0.85–1.12)	0.74	1.41 (1.23–1.60)	<0.001
Model 2	1.00 (reference)	1.05 (0.92–1.21)	0.42	1.53 (1.35–1.76)	<0.001
Model 3	1.00 (reference)	0.97 (0.85–1.12)	0.69	1.36 (1.19–1.56)	<0.001

Cox proportional hazard regression models for IBS were performed.

Model 1: Unadjusted.

Model 2: Model 1 + age, sex.

Model 3: Model 2 + ethnicity, BMI, Index of Multiple Deprivation, sleep duration, smoking status, alcohol drinker status, anxiety or depression.

IBS, Irritable bowel syndrome; BMI, Body mass index.

In the subgroup analysis (Table 3), we found that night shift work increased the risk of IBS onset, and this association remained stable across subgroups stratified by gender (male or female), age (≥50 years or <50 years), sleep duration (≥7 h or <7 h), and mental health status (anxiety and depression). Notably, across BMI subgroups, we found that among individuals with a BMI ≥ 25 kg/m^2^, those who always worked night shifts had a higher risk of developing IBS, with an HR of 1.49 (1.28–1.73).

**Table 3:** Subgroup analysis of the association between night shift work and irritable bowel syndrome.

Variables	Count	Sometimes/usually night shifts	*p*-value	Always night shifts	*p*-value	*p* for interaction
Sex						0.99
Female	135,448	1.07 (0.9–1.29)	0.43	1.54 (1.29–1.84)	<0.001	
Male	131,157	1.05 (0.85–1.3)	0.65	1.56 (1.27–1.9)	<0.001	
Age (years)						0.86
≥50	171,980	0.98 (0.81–1.18)	0.82	1.44 (1.21–1.72)	<0.001	
<50	94,625	0.95 (0.78–1.17)	0.65	1.33 (1.09–1.63)	0.005	
Anxiety or depression						0.62
No	183,946	0.93 (0.76–1.13)	0.44	1.42 (1.18–1.71)	<0.001	
Yes	82,659	1.06 (0.88–1.29)	0.53	1.43 (1.18–1.73)	<0.001	
Sleep duration (h)						0.76
≥7	198,241	0.93 (0.78–1.11)	0.42	1.3 (1.09–1.56)	0.004	
<7	68,364	0.99 (0.8–1.24)	0.95	1.41 (1.16–1.73)	0.001	
BMI (kg/m^2^)						0.003
≥25	175,326	1.1 (0.95–1.28)	0.22	1.49 (1.28–1.73)	<0.001	
<25	91,279	0.66 (0.49–0.9)	0.008	1.15 (0.87–1.53)	0.33	

Never/rarely night shifts were set as the reference.

BMI, Body mass index.

In the sensitivity analysis (Table 4), it was observed that after excluding new IBS cases within the first year of enrollment and including residential address and physical activity as covariates, the results remained consistent with those of the primary analysis. The hazard ratios were 1.39 (1.21–1.60) and 1.38 (1.17–1.61), respectively.

**Table 4:** Sensitivity analysis of the association between night shift work and irritable bowel syndrome.

Variables	Never/rarely night shifts	Sometimes/usually night shifts	*p*-value	Always night shifts	*p*-value
Primary analysis	1.00 (reference)	0.97 (0.85–1.12)	0.69	1.36 (1.19–1.56)	<0.001
Sensitivity analysis 1	1.00 (reference)	0.97 (0.84–1.12)	0.72	1.39 (1.21–1.60)	<0.001
Sensitivity analysis 2	1.00 (reference)	1.04 (0.89–1.21)	0.63	1.38 (1.17–1.61)	<0.001

Cox proportional hazard regression models for IBS were performed.

Primary analysis: Model 1 + age, sex, ethnicity, BMI, Index of Multiple Deprivation, sleep duration, smoking status, alcohol drinker status, anxiety or depression.

Sensitivity analysis 1: excluded new IBS cases within the first year of enrollment.

Sensitivity analysis 2: primary analysis + urban/rural residence, physical activity.

IBS, Irritable bowel syndrome; BMI, Body mass index.

## 4 Discussion

In this large-scale cohort study using UK Biobank, 266,605 participants were included. We found that compared with those who never/rarely worked night shifts, people who always worked night shifts had a higher risk of developing IBS. Secondly, this result remained robust after adjusting for covariates and excluding new IBS cases in the first year. Finally, subgroup analysis showed that the increased risk of IBS due to night shift work persisted among people of different genders, ages, sleep durations, and mental health statuses. It is worth highlighting that compared to those who never/rarely worked night shifts, the higher risk of developing IBS among individuals who always worked night shifts was more pronounced among individuals with a BMI ≥ 25 kg/m^2^.

To our knowledge, this is the first research investigating the association between night shift work and the risk of incident irritable bowel syndrome using a large-scale population-based cohort. Previous studies have explored the relationship between shift work and IBS. For instance, Hye in Kim and Borko Nojkov found that shift-working nurses had a higher prevalence of IBS compared to day-shift nurses (24, 25). While Hamid Reza Saberi reported higher rates of gastrointestinal symptoms among rotating-shift nurses than day-shift nurses (30). These findings are consistent with our conclusions. However, these earlier studies had generally smaller sample sizes and were predominantly focused on specific occupational groups, thereby limiting the reliability and generalizability of their conclusions.

The specific mechanisms by which night shifts increase the risk of IBS remain unclear. We hypothesize that potential mechanisms linking night shift work to elevated IBS risk may include disruption of the body’s normal circadian rhythm through altered sleep–wake cycles, leading to gut microbiota dysbiosis (31). The circadian misalignment may activate pro-inflammatory cytokines and promote low-grade intestinal inflammation, thereby contributing to IBS pathogenesis (32, 33). Additionally, melatonin, a hormone critical for both sleep regulation and gastrointestinal protection (34, 35), may play a mediating role. As night shift work suppresses melatonin secretion (36). Notably, melatonin supplementation has shown therapeutic potential in alleviating symptoms and improving the quality of life for IBS patients (37, 38). Shift workers’ dietary habits have changed, they prefer high-fat and high-sugar foods, along with irregular eating patterns, which may increase the risk of IBS (39, 40). Disruption of the circadian rhythm impairs normal gastrointestinal motility, thereby contributing to the development of IBS (41).

The elevated IBS risk associated with night shifts was observed in the subgroup with BMI ≥ 25 kg/m^2^, which aligns with previous research findings (42). This phenomenon may be related to accelerated intestinal transit time in obese patients (43). Individuals with high BMI consume more ultra-processed foods (44), and high consumption of ultra-processed foods increases the risk of developing IBS (45). Individuals with obesity exhibit alterations in their gut microbiota (46), and their adipose tissue can induce systemic low-grade inflammation (47). People with a low BMI engage in more physical activity, which may be associated with a reduced risk of developing IBS (48, 49). These factors may explain why the association between night shift work and IBS risk differs across BMI groups (50, 51).

It is worth noting that the risk of developing IBS only increased among those who always worked night shifts. This finding aligns with the impact of shift work, particularly permanent night shifts, on dyslipidemia (52). We hypothesize that the cumulative effects of night shifts on the body, mediated through the aforementioned mechanisms, intensify with a higher frequency of night shifts. As the intensity of stressors increases, the resulting harm becomes more severe, ultimately contributing to the onset of disease (53).

We did not observe significant effect modifications by shift work patterns on the risk of IBS onset across different sleep duration groups. Previous studies have indicated a consistent positive association between self-reported sleep disturbances and gastrointestinal symptoms of IBS (54). In our study, sleep duration was self-reported by participants, and covariates related to sleep such as insomnia and snoring were not included. Future research could consider incorporating a more comprehensive set of sleep-related covariates to further elucidate the role of sleep in the relationship between shift work and the risk of IBS development.

There are several advantages in our study. First of all, this is the first large-scale, long-term follow-up cohort study to investigate the association between night shift work and the risk of developing IBS. Secondly, our study boasts an exceptionally large sample size with strong data representativeness. We included a total of 266,605 participants from the UK Biobank, which encompasses individuals with diverse characteristics, including different ages, genders, and socioeconomic backgrounds, ensuring high generalizability. Finally, the UK Biobank data was systematically collected and recorded by professionals using standardized protocols and procedures, which minimizes measurement errors and biases, thereby enhancing the authenticity and accuracy of the findings.

There are several limitations. Firstly, this is an observational study and cannot establish a causal relationship between night shift work and IBS. Rigorously designed randomized controlled trials or additional large-scale cohort studies are required for verification. Secondly, although we controlled for confounding factors such as age, gender, and sleep duration, potential residual confounders like dietary factors, marital status, occupational type, and psychosocial factors might still exist. Moreover, due to data limitations, we were unable to measure the exposures as accurately as laboratory parameters or identify specific subtypes of IBS. Besides, the use of ICD-10 codes for case definition in the UK Biobank may introduce healthy volunteer selection bias and under-diagnosis bias (55). And we were unable to accurately distinguish between IBS subtypes. Finally, as UK Biobank participants are primarily of European descent, the observed association between night shift work and IBS risk may not be directly generalized to populations in the Americas, Asia, or other areas, necessitating region-specific cohort investigations.

## 5 Conclusion

Through this cohort study, we found that individuals who always worked night shifts exhibited a higher risk of developing IBS compared to those who never/rarely engaged in night shift work. Our findings recommend that individuals at high risk for IBS or those diagnosed with IBS should avoid night shift work and prioritize adherence to a normal circadian rhythm. The other effects of night shift work on the body and their underlying mechanisms still require further research to investigate.

## Data Availability

Publicly available datasets were analyzed in this study. This data can be found at: https://www.ukbiobank.ac.uk/ Project ID:99732.
