# Supplementary material for: Night shift work increases the risk of developing irritable bowel syndrome: a prospective cohort study in the UK Biobank
Source: Front Public Health. 2025 Oct 16;13:1651752. doi: 10.3389/fpubh.2025.1651752 (PMC12571640; doi:10.3389/fpubh.2025.1651752)
Supplement: Supplementary file 1 [file Table_1.docx]

Supplementary Table 1 Source of report of irritable bowel syndrome

| Source | Number |
| --- | --- |
| Primary care only | 1566 (30.01%) |
| Primary care and other source(s) | 231 (4.43%) |
| Hospital admissions data only | 3002 (57.53%) |
| Hospital admissions data and other source(s) | 33 (0.63%) |
| Self-report only | 318 (6.09%) |
| Self-report and other source(s) | 68 (1.30%) |
